# Measurement data on domestic hot water consumption and related energy use in hotels, nursing homes and apartment buildings in Norway

**DOI:** 10.1016/j.dib.2021.107228

**Published:** 2021-06-15

**Authors:** Harald Taxt Walnum, Åse Lekang Sørensen, Karolina Stråby

**Affiliations:** Department of Architectural Engineering, SINTEF, P.O. Box 124 Blindern, Oslo 0314, Norway

**Keywords:** Domestic hot water, Water consumption, Energy use, Distribution losses, Hot water circulation, Peak flow rate, Field measurements

## Abstract

The data article describes detailed measurements of domestic hot water (DHW) consumption in 12 Norwegian buildings. Included in this study are 4 hotels, 4 nursing homes, and 4 apartment buildings in the greater Oslo region. Flow and temperature measurements were performed on the DHW production system in each building's heating plant, for a duration of at least 6 weeks. The measurements were conducted with an interval of 1 s, and then averaged for 2 s before analysis in order to reduce data noise and measurement uncertainties. The data set includes flow rates, temperatures, energy for consumed hot water (CHW) and distribution losses in the hot water circulation (HWC). Reuse potentials consist of analyses related to flow rates, energy use and distribution losses, e.g. in peak flow rates analysis or DHW energy flexibility simulations. The measurements were performed within the research project “Energy for domestic hot water in the Norwegian low emission Society” (VarmtVann2030).

## Specifications Table

**Table:** 

Subject	Renewable Energy, Sustainability and the Environment
Specific subject area	Domestic hot water (DHW) consumption and energy use in buildings
Type of data	CSV files Tables
How data were acquired	Flow and temperature measurements were performed on the main supply for 12 buildings. • Clamp-on ultrasonic flow meters were used for flow measurement (FLUXUS F601 [1]). • Type-*T* thermocouples where mounted on the pipe wall (TE Wire & Cable [2]). • Data logger (Squirrel 2020 [3]) • Data treatment in python/pandas [4]
Data format	Raw
Parameters for data collection	• Measurements on DHW system in 12 buildings: Hotels (4), nursing homes (4), and apartment buildings (4). • Temperatures and flow rates are measured in the heating central, with an interval of 1 s and averaged to 2 s. • Energy for consumed hot water (*Q*CH*W*) and energy for hot water circulation (*Q_HWC_*) is calculated.
Description of data collection	• At each location, the measurement equipment was installed for a period of minimum 6 weeks. • Measurements with an interval of 1 s are averaged to 2 s, to reduce uncertainty / noise in the measurements. • Data is presented with time steps of 2 s and 1 h.
Data source location	Buildings: Hotels (4), nursing homes (4), and apartment buildings (4) City/Town/Region: Greater Oslo Region (Oslo, Drammen, Lillestrøm) Country: Norway
Data accessibility	Repository name: Mendeley Data Data identification number: 10.17632/m3xy22pf4j.1 Direct URL to data: http://dx.doi.org/10.17632/m3xy22pf4j.1
Related research article	The data article is related to research articles listed on the homepage of the project VarmtVann2030 [5], such as: H. Taxt Walnum, Å.L. Sørensen, B. Ludvigsen, D. Ivanko, Energy consumption for domestic hot water use in Norwegian hotels and nursing homes, IOP Conf. Ser. Mater. Sci. Eng. 609 (2019). 10.1088/1757-899X/609/5/052020[6]

## Value of the Data

• While buildings are becoming more energy efficient, the share of DHW energy is increasing. DHW energy use is often identified as a main source of flexible energy use in buildings, due to the DHW storage tanks. It is becoming increasingly important to understand the energy use and energy losses related to DHW.
• The datasets provide temperature and flow rate measurements with high (2 s) resolution in hotels, nursing homes, and apartment buildings. Energy for consumed hot water and energy for hot water circulation is described with high and hourly resolution. Researchers, energy analysts, building owners and industrial players can benefit from the datasets, analysing DHW flow rates, energy use and distribution losses. Detailed data on DHW consumption is important background for development of standards and directives, e.g. related to pipe dimensioning and energy labels.
• The datasets can be an important input for various analyses related to flow rates, energy use and distribution losses, e.g. in peak flow rates analysis or DHW energy flexibility simulations.
• Data from Norwegian buildings can be useful for studies related to differences in energy consumption between countries and societies.

## 1 Data Description

### 1.1 Description of buildings and measurement periods

Measurements are performed in 12 buildings: 4 hotels (HO), 4 nursing homes (NH), and 4 apartment buildings (AB). The main parameters describing the buildings are shown in Table 1.

**Table 1:** Main building parameters.

Building ID	Area [m^2^]	# Rooms or apt.	Distribution heating	Comments to distribution system	# Basin mixers	# Kitchen mixers	# Shower mixers	# Bath mixers	Measurement period
HO1	21,278	434	Circulation	Poorly balanced, only sections of the building had circulating water.	514	31	316	173	13.03.2018– 25.04.2018
HO2	24,500	355	Circulation	Circulation only covers part of the building. The rest is covered by electric heat tracing, which is not included in calculations.	527	30	275	105	24.08.2018– 07.10.2018
HO3	4934	139	Circulation	Circulation seems to work as intended.	145	5	135	0	24.08.2018– 07.10.2018
HO4	7440	151	Circulation	Circulation seems to work as intended.	166	25	153	10	30.03.2018– 15.08.2018
NH1	11,618	148	Circulation	Circulation seems to work as intended. Most likely uninsulated pipe-in-tube systems in shafts.	175	121	158	0	26.01.2018– 22.02.2018
NH2	3327	52	Electric heat tracing	Electric heat tracing not included in measurements.	53	4	52	0	31.05.2018– 11.07.2018
NH3	6774	50	Circulation	Circulation plugged and short-circuited just outside heating central.	55	5	55	0	26.05.2018– 11.07.2018
NH4	10,081	96	Circulation	Circulation seems to work as intended. New building.	123	92	105	0	16.01.2019– 06.03.2019
AB1	4400	96	Circulation	9-floor building with circulation in basement only.	96	96	96	0	20.10.2018– 09.12.2018
AB2	2700	56	Circulation	Recently renovated plumbing system with circulation branches up in every shaft.	56	56	56	0	23.10.2018– 09.12.2018
AB3	3752	56	Circulation	4-floor building with circulation in basement only.	56	56	56	0	16.01.2018– 06.03.2018
AB4	5100	86	Circulation	Due to small and old tubes HWC data have poor measurement quality with high uncertainty and are therefore not included.	86	86	86	0	30.03.2019– 18.08.2019

All buildings except NH2 have hot water circulation (HWC) systems, where DHW is permanently circulated in pipes to keep the water hot, compensating for heat losses. However, there were large differences in the layout and several of them do not work as intended. Either they were not properly balanced, so that the water only circulated in a limited part of the system, or blinded after some renovation work on the system. See Table 1 for details. This makes it difficult to compare the losses.

HO1 and HO2 are built according to similar specifications, and are both typical conference hotels, but HO1 does have higher share of non-business guests. HO3 is a more compact city hotel, without restaurant and conference halls. HO4 is also a city hotel, but with a restaurant and large kitchen facilities.

The main difference between the nursing homes is the room density (number of resident rooms per total area). NH3 has a lower room density than the other two buildings. In addition, NH3 has bypassed a large part of the circulation system. In general, most residents in nursing homes have their own room with separate bathroom. Most hot food is made at centralized kitchens and transported to the nursing homes.

For the apartment buildings, most of the apartments in AB1 and AB2 have 1 bedroom, the apartments in AB3 have 2 bedrooms, and the apartments in AB4 have from 2 to 3 bedrooms. AB1 and AB2 are social housing, owned and managed by the state to provide affordable housing for people who need it. AB3 and AB4 are housing cooperatives with privately owned apartments.

### 1.2 Dataset 1: flow, temperature, and energy data with 2 s resolution

Dataset 1 describes flow, temperature, and energy data with 2 s resolution. The dataset is divided in 12 csv-files, one for each building. The csv-files are accessible from [7], labeled [building ID]_1. Table 2 shows the parameters available for each of the buildings. Some general comments to the data:

• Data timestamps are given in Central European Time (CET), which is GMT +1. Daylight saving time (DST) applies.
• The data is unfiltered (except for the 2 s average) and especially the flow measurements therefore have significant noise.
• In some buildings systems had malfunctioning non-return valves, resulting in periods with negative flow rates and increased cold-water temperatures.

**Table 2:** Description Dataset 1: Available flow, temperature, and energy data with 2 s resolution.

Time		Start-time for measurement (CET, DST applies). Format yyyy-MM-dd hh:mm:ss												
*Label*	*Unit*	*Description*	HO1	HO2	HO3	HO4	NH1	NH2	NH3	NH4	AB1	AB2	AB3	AB4
T_cw	°C	Cold water inlet temp.	x	x	x	x	x	x	x	x	x	x	x	x
T_hw	°C	Hot water for distribution temp.	x	x	x	x	x	x	x	x	x	x	x	x
T_hwc	°C	Return circulation temp.	x	x	x	x	x	NA	x	x	x	x	x	x
V_hwc	l/s	HWC return flow rate	x	x	x	x	x	NA	x	x	x	x	x	-
V_chw	l/s	Consumed hot water flow rate	x	x	x	x	x	x	x	x	x	x	x	x
P_chw	W	Power for consumed hot water	x	x	x	x	x	x	x	x	x	x	x	x
P_hwc	W	Power for HWC heat losses	x	x	x	x	x	NA	x	x	x	x	x	-

### 1.3 Dataset 2: energy data with hourly resolution

Dataset 2 describes energy data with hourly resolution. The dataset is divided in 12 csv-files, one for each building. The csv-files are accessible from [7], labeled [building ID]_2. Table 3 shows the parameters available for each of the buildings.

**Table 3:** Description Dataset 2: Available energy data with hourly resolution.

Time		Start-time for measurement (CET, DST applies). Format yyyy-MM-dd hh:mm												
*Label*	*Unit*	*Description*	HO1	HO2	HO3	HO4	NH1	NH2	NH3	NH4	AB1	AB2	AB3	AB4
Q_chw	kWh	Energy for consumed hot water	x	x	x	x	x	x	x	x	x	x	x	x
Q_hwc	kWh	Energy for HWC heat losses	x	x	x	x	x	NA	x	x	x	x	x	-

## 2 Experimental Design, Materials and Methods

### 2.1 Measurement equipment

Detailed measurements of water flow and temperature were performed on the DHW production system in each building, for a duration of approx. 6 weeks. The measurements were conducted with an interval of 1 s, and then averaged for 2 s to reduce measurement uncertainties and noise. In order to avoid modifications to the water installations, non-intrusive clamp-on ultrasonic flow meters and Type-*T* thermocouples mounted on the pipe outer wall were used. The flow meters have a specified accuracy of 1.6% of reading ±0.01 m/s [1], and the Type-T thermocouples have an error specified as maximum of 1.0 °C or 0.75% above 0 °C [2]. For the flow meters, pipe diameter and thickness are needed as input. For copper piping thickness was measured with an accompanying tool, while for alupex piping, manufacturer data was applied. Thermocouples were mounted on the outer pipe wall and fixed with aluminium tape, and then insulation was added on the outside. All data was logged with a local logger [3], to avoid issuse with wireless data transfer and connection.

### 2.2 Measurement setup

**Fig. 1:**
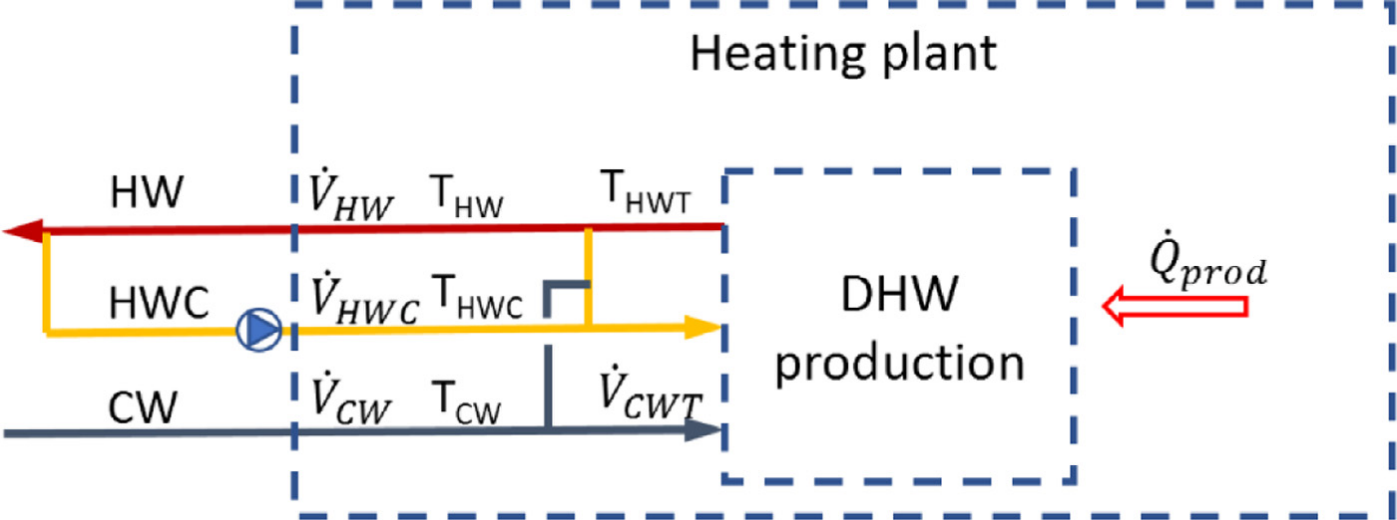
Principle drawing of DHW heating plants with typical measuring points (based on Fig. 1 in [6]), where DHW is Domestic hot water, HW is DHW inclusive HWC, HWC is hot water circulation, HWT is hot water from production, CW is cold water for DHW (same as consumed DHW), CWT is cold water to DHW production (not mixing valve), $\dot{V}$ is Flow rate [l/s], T is temp. [°C], and ${\dot{Q}}_{prod}$ is energy to DHW production [kWh] (not included with article).

There are variations in how DHW systems in Norway are designed, both in regard to energy sources, but also with respect to system layout. Fig. 1 shows a principle drawing of how most heating plants are built, with typical measuring points used in the DHW measurements. When possible, all measuring points shown are logged. However, in many cases, the pipe sections between junctions are too short or there are other branches that influence the measurements. Tables 2 and 4 show data available for the individual buildings, where the values in Table 2 are available with the article. The values are either measured (Table 2: T_cw, T_hw, T_hwc, V_hwc, V_chw; Table 4: T_hwt, V_cw, V_cwt, V_hw) or calculated (Table 2: V_chw, P_chw, P_hwc). The calculations are presented in the next section.

**Table 4:** Measurements performed for each of the buildings, in addition to the data presented in Table 2.

*Label*	*Unit*	*Description*	HO1	HO2	HO3	HO4	NH1	NH2	NH3	NH4	AB1	AB2	AB3	AB4
T_hwt	°C	HWT temp.		x	x	x	x	x	x		x	x	x	x
V_cw V_chw	l/s	CW flow rate for DHW, same as CHW flow rate.	x		x	x						x		x
V_cwt	l/s	Cold water flow rate to DHW production (not mixing valve)		x			x	x	x		x			
V_hw	l/s	DHW flow rate, inclusive HWC		x	x	x	x	x	x	x	x	x	x	

### 2.3 Calculations

#### 2.3.1 Water for hot water production

Consumed DHW flow rate (${\dot{V}}_{CHW}$) is the same as cold water flow rate into the production unit (${\dot{V}}_{CW}$). Some buildings had short pipe sections where the flow branches off. This made it challenging to achieve accurate measurements of ${\dot{V}}_{CW}$ without interference from adjacent pipe runs. For these buildings, ${\dot{V}}_{CW}$ was calculated according to Eq. (1).

(1) $${\dot{V}}_{CHW}={\dot{V}}_{CW}={\dot{V}}_{HW}-{\dot{V}}_{HWC}$$

#### 2.3.2 Power and energy flows

Power for consumed hot water (${\dot{P}}_{CHW}$) and heat loss (${\dot{P}}_{HWC}$) are calculated according to Eqs. (2) and (3), respectively. *h(T)* denotes the specific enthalpy of water at temperature T, and *ρ* is the density of the water at the temperature of which the flow rate is measured. The hourly energy flows (Q) are calculated as the mean of the power within each hour.

(2) $${\dot{P}}_{CHW}=\frac{{\dot{V}}_{CHW}}{\rho }*\left(h(T_{HW}\right)-h\left(T_{CW}\right))$$

(3) $${\dot{P}}_{HWC}=\frac{{\dot{V}}_{HWC}}{\rho }*\left(h(T_{HW}\right)-h\left(T_{HWC}\right))$$

## Ethics Statement

Data are provided with consent from the building owners.

## CRediT Author Statement

**Harald Taxt Walnum**: Conceptualization, Methodology, Investigation, Data Curation, Writing- Original draft preparation; **Åse Lekang Sørensen**: Conceptualization, Writing- Original draft preparation; **Karolina Stråby**: Investigation, Writing- Original draft preparation.

## Declaration of Competing Interest

The authors declare that they have no known competing financial interests or personal relationships which have or could be perceived to have influenced the work reported in this article.
